# Identification of Constituents and Exploring the Mechanism for Toutongning Capsule in the Treatment of Migraine

**DOI:** 10.1155/2022/5528845

**Published:** 2022-01-15

**Authors:** Xia Du, Zhibiao Di, Yang Liu, Wenbing Zhi, Yuan Liu, Hong Zhang, Feng Liu

**Affiliations:** ^1^Institute of Traditional Chinese Medicine, Shaanxi Academy of Traditional Chinese Medicine, Xi'an, Shaanxi 710003, China; ^2^Center for Post-Doctoral Studies, China Academy of Chinese Medical Sciences, Beijing 100700, China; ^3^Shaanxi Institute of International Trade &Commence, Xi'an 712046, China; ^4^Shaanxi Buchang Pharmaceutical Co. Ltd, Xi'an 710075, China

## Abstract

Toutongning capsule (TTNC) is an effective and safe traditional Chinese medicine used in the treatment of migraine. In this present study, a multiscale strategy was used to systematically investigate the mechanism of TTNC in treating migraine, which contained UPLC-UESI-Q Exactive Focus network pharmacology and experimental verification. First, 88 compounds were identified by the UPLC-UESI-Q Exactive Focus method for TTNC. Then, the target fishing for these compounds was performed by means of an efficient drug similarity search tool. Third, a series of network pharmacology experiments were performed to predict the key compounds, targets, and pathways. They were protein-protein interaction (PPI), KEGG pathway enrichment analysis, and herbs-compounds-targets-pathways (H-C-T-P) network construction. As a result, 18 potential key compounds, 20 potential key targets, and 6 potential signaling pathways were obtained for TTNC in treatment with migraine. Finally, molecular docking and experimental were carried out to verify the key targets. In short, the results showed that TTNC is able to treat migraine through multiple components, multiple targets, and multiple pathways. This work may provide a theoretical basis for further research on the molecular mechanism of TTNC in the treatment of migraine.

## 1. Introduction

Migraine is a common chronic and paroxysmal neurological disease. The most characteristic symptoms associated with migraine include nausea, emesis, photophobia, and phonophobia. In addition, patients tend to have a variety of other neurological symptoms, such as dizziness, tinnitus, and cognitive impairment [1, 2]. Epidemiological survey results showed that the overall incidence of migraine in the general population was 12%, and the annual cost of migraine was estimated to be more than US$20 billion. Plus, the WHO ranked migraine as the third most prevalent medical condition and the second most disabling neurological disorder in the world [3–5]. In recent years, many diagnostic guidelines for migraine have shown up and played an important role in its prevention and treatment. However, due to the high incidence of migraine, high disability, high missed diagnosis rate, and low control rate, the current diagnosis and treatment outcome of migraine are still not ideal. Acute treatment of migraine includes the use of drug therapy, from ergots and triptans to nonsteroidal anti-inflammatory drugs [6, 7]. It is known that triptans have been the leading evidence-based, acute therapies for migraine for the past 20 years, including sumatriptan, naratriptan, rizatriptan, zolmitriptan, eletriptan, almotriptan, frovatriptan, and sumatriptan/naproxen combination [8, 9]. These FDA-approved drugs affect serotonin and have proven efficacy in the acute treatment of migraine. Nevertheless, most of all these drugs were short-acting. Also, they were contraindicated in coronary artery disease, uncontrolled hypertension, and Prinzmetal angina. In addition, preventive treatment medications can reduce the frequency, severity, and duration of attacks in people with frequent migraine, such as beta-blockers (propranolol and metoprolol), calcium channel blockers (flunarizine), antiepileptics (sodium valproate, topiramate, lamotrigine, and gabapentin), and antidepressants (amitriptyline, fluoxetine, and sertraline) [10–12]. Depressingly, the long-term use of these drugs may result in adverse effects such as lethargy, fatigue, anxiety, depression, and muscle soreness.

Traditional Chinese medicine (TCM) has been widely used in the treatment of migraine for more than 2000 years. Migraine belongs to the category of Shou Feng or Nao Feng in TCM, which has been recorded as early as in the Yellow Emperor's Medicine Classic (Chinese name in pinyin Huang Di Nei Jing), a classical literature from the Han Dynasty (AD 25–220). In TCM, “wind, heat, phlegm, and blood stasis” are considered to be the main pathogenic factor of migraine. In this regard, the critical points were extinguishing wind and resolving phlegm activate blood and relieving stasis in the treatment of migraine. Toutongning capsule (TTNC) functions calming wind, clearing phlegm, removing blood stasis, and relieving pain. Hence, Toutongning capsule is frequently used in the treatment of migraine in clinical and has a significant effect. The components of the formula include Smilacis Glabrae Rhizoma, Gastrodiae Rhizoma, Polygoni Multiflori Radix, Angelicae Sinensis Radix, Saposhnikoviae Radix, and Scorpio. Among these herbs, Gastrodiae Rhizoma is able to calm the liver to stop endogenous wind and combined with Smilacis Glabrae Rhizoma can pacify the liver to subdue yang. Polygoni Multiflori Radix has the effect of replenishing lean and nourishing blood. Angelicae Sinensis Radix can nourish blood and activate blood. Saposhnikoviae Radix is able to dispel wind and eliminate dampness. Scorpio can break blood, expel stasis, and free the collateral vessels to relieve pain. The results of evidence-based medicine research showed that the effective rate of TTNC was 72.5%, with a prevention rate of 86.5%. No adverse events took place for the treatment of 400 patients with primary migraine by a randomized, double-blind, placebo-controlled multicenter clinical trial [13]. At present, most research studies on TTNC tended to focus on clinical research. There are few studies on the molecular mechanism based on the multiple components and multiple targets of TTNC.

**Figure 1:**
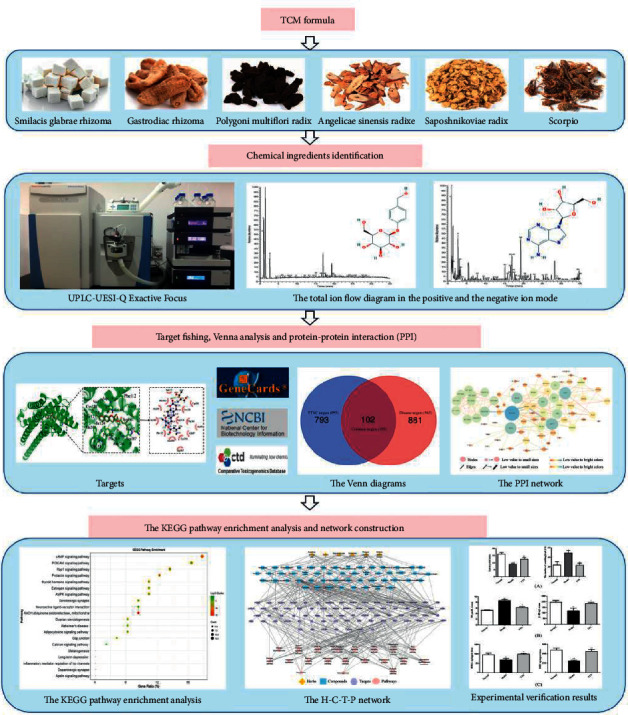
The detailed flowchart for TTNC in the treatment of migraine.

In this present study, an integrated strategy was used to investigate the mechanism of TTNC in treating migraine, which contained UPLC-UESI-Q Exactive Focus and network pharmacology. The UPLC-UESI-Q Exactive Focus method was adopted to identify the chemical composition for TTNC. The technologies of network pharmacology [14, 15] were performed for clarifying the potential mechanism of action for TTNC in treatment with migraine, such as the target fishing, protein-protein interaction (PPI), KEGG pathway enrichment analysis, and herbs-compounds-targets-pathways (H-C-T-P) multidimensional network construction (Figure 1). This present study may provide a theoretical basis for further research on the molecular mechanism of TTNC in the treatment of migraine.

## 2. Materials and Methods

### 2.1. The Chemical Composition Analysis by UPLC-UESI-Q Exactive Focus

#### 2.1.1. Chemicals and Materials

Acetonitrile (HPLC-grade) and methyl alcohol (HPLC-grade) were obtained from Fisher Scientific Company. The MS-grade formic acid was obtained from Sigma Aldrich (Germany) Trading Co., Ltd. Toutongning capsule (TTNC) (batch no. 190312) was provided by Buchang Pharmaceuticals (Xi'an, China).

#### 2.1.2. UPLC-UESI-Q Exactive Focus Conditions

The instrument used for the chromatographic separation was a Thermo Scientific Q Exactive Focus system coupled with the Thermo Scientific Ultimate 3000 system [16, 17]. Separation was performed on an Accucore aQ C18 column (150 mm × 2.1 mm, 2.6 *μ*m). In addition, column temperature was maintained at 30°C. The mobile phase consisted of two solvents A (0.1% formic acid in water) and B (methyl alcohol) at a flow rate of 0.30 mL/min. A linear gradient elution of A and B was used according to the following program (0–10 min, 5–25% B; 10–25 min, 25–60% B; 25–30 min, 60% B; 30–35 min, 60–95% B; 35–37 min, 95–5% B; and 37–40 min, 5% B). The injection volume was set 3 *μ*L. Liquid chromatography HESI-MS analysis was conducted by means of the positive and negative electrospray model. The mass spectral scan ranges from 80 Da to 1200 Da. The spray voltage was set at 3.5 kV. Besides, the other conditions are shown as follows: capillary temperature, 320°C; Aux gas heater temperature, 350°C; sheath gas flow rate, 40 arb; Aux gas flow rate, 10 arb; S-lens RF level, 50.0 V; resolution, full MS 70000, dd-MS2 35000; and scanning mode, full MS-dd-MS2.

### 2.2. The Mechanism of Toutongning Capsule (TTNC) in the Treatment of Migraine by Means of Network Pharmacology

#### 2.2.1. Target Fishing of the Chemical Composition for TTNC

The potential targets of the chemical composition by UPLC-UESI-Q Exactive Focus for TTNC were predicted in an encyclopedia of traditional Chinese medicine (ETCM) platform using MedChem Studio (version 3.0), which is an efficient drug similarity search tool to identify the similarity between the known drugs in the DrugBank database with the tested compounds [18, 19]. The similarity is manifested by the Tanimoto score, and the Tanimoto score is in the range of (0, 1), where “0” denotes completely different structures between ingredients and known drugs, and “1” denotes identical structures of two components. In this present study, the threshold of the Tanimoto scores was set to 0.75.

#### 2.2.2. The Targets Related to the Migraine

The targets related to the migraine were collected from the GeneCards database. The targets with relevance score above the average were regarded as the migraine targets. Relevance score is the Novoseek score of the relevance of the disease to this gene based on their literature text-mining algorithms [20]. All targets were converted by means of UniProt database and corrected to the official shorthand [21].

#### 2.2.3. The Targets of the Chemical Composition for TTNC in the Treatment of Migraine

The nonhuman targets were deleted from all the component targets and disease-causing genes. After that, Venn online tool free software (https://bioinformatics.psb.ugent.be/webtools/Venn/) was used to screen the overlap of disease-causing genes and component targets for TTNC.

#### 2.2.4. Protein-Protein Interaction (PPI) Network Construction and Core Target Screening

The targets were introduced into the online system public database STRING (Search Tool for Known and Predicted Protein-Protein Interactions, version 11.0, https://string-db.org/) to obtain the interaction between proteins and predicted proteins [22]. The protein type was set to “*Homo sapiens*,” and the minimum interaction threshold with the highest confidence was −0.900, and the targets with poor connection of the primary network were removed. After then, the PPI network was constructed using Cytoscape 3.7.2, and the network topological properties were analyzed for each node using the CytoNCA plugin for Cytoscape 3.7.2, such as degree centrality (DC), betweenness centrality (BC), and closeness centrality (CC) [23].

#### 2.2.5. KEGG Pathway Analysis

The biological functions and participated pathways of the candidate drug targets have been investigated according to KEGG (Kyoto Encyclopedia of Genes and Genomes) pathway analysis by means of DAVID (Database for Annotation Visualization and Integrated Discovery) [24, 25].

#### 2.2.6. The Herbs-Compounds-Targets (H-C-T-P) Network Construction and Analysis

The H-C-T-P network was constructed for TTNC in the treatment of migraine by Cytoscape 3.7.2. The network topological properties, including DC, BC, and CC, were analyzed by the NetworkAnalyzer plugin in the software to explore the key compounds and targets for TTNC in the treatment of migraine.

### 2.3. Molecular Docking

Molecular docking was performed to investigate the interactions between some key components and targets. The structures of the key components were obtained from the PubChem database (https://pubchem.ncbi.nlm.nih.gov). The structures of the targets were obtained from the PDB database (https://www.pdb.org). The docking simulation was performed by means of AutoDock 4.2.6 software. During the docking calculations, hydrogen atoms and Gasteiger charges were added to the protein using the automated docking tool. The auxiliary program AutoGrid was used to set the docking boxes. These docking boxes were defined according to the crystal structures of complex of the proteins with known ligands. The Lamarckian genetic algorithm (LGA) was adopted for each docking progress.

### 2.4. Experimental Verification

#### 2.4.1. Animals

Sprague-Dawley (SD) rats (*n* = 24), 6 weeks, weighing 180–220 g, were purchased from Xi'an Jiaotong University; the certificate number was SCXK2017-003. The rats were fed with a standard pellet diet and tap water ad libitum, and they were housed and maintained in 12 h light/darkness with standard humidity and temperature in the laboratory. The principles of laboratory animal care guidelines, approved by the Animal Ethics Committee at the Shaanxi Academy of Traditional Chinese Medicine, were strictly followed.

#### 2.4.2. Establishment and Grouping of the Migraine Rat Model

After an environmental adaptation period of 7 days, the rats were randomly divided into three groups, including the control group, model group, and TTNC group (640 mg/kg/d). The TTNC group was given intragastric administration for 7 days, and the control group and model group were given equal volume normal saline. After the last administration of 30 min, the rats were subcutaneously injected with nitroglycerin injection 10 mg/kg (Beijing Yimin Pharmaceutical Co., Ltd.) in the TTNC group and model group, and the control group was injected subcutaneously with equal volume of normal saline. The number of scratching and other behavioral manifestations of rats were recorded within 1 h after injection. When the rats showed irritability such as red ears, frequent head scratching, and cage climbing, it indicates that the modeling was successful. The rats were anesthetized with pentobarbital, blood was taken from the abdominal aorta, standing 2 h, centrifugation at 1000 rpm for 10 min, and then blood serum was obtained. At the same time, the brainstem was isolated and brain tissue was obtained and collected in the EP tube. Blood serum and brain tissue were stored at −80°C until used.

#### 2.4.3. Preparation of Serum and Tissue Homogenate

Rat blood was taken from the abdominal aorta after anesthetized with pentobarbital, and serum was obtained. The brainstem was isolated, and the tissue was collected in EP tubes. Both serum and brain tissue were stored at −80°C until used. A certain amount of tissue was cut and weighed. Ten times the amount of normal saline was added for further grinding (Haoyuan Technology Co., Ltd., China). After centrifuging twice at 4°C, the supernatant was collected, and the protein quantification was determined by the BCA protein concentration assay kit (Beyotime, Shanghai, China).

#### 2.4.4. Indexes Detection in Serum and Tissue

According to the manufacturer's protocols, the content of *β*-EP in blood serum was detected by commercial enzyme-linked immunosorbent assay ELISA kits. The nitrate reductase method was used to detect the level of NO in serum, strictly following NO one-step detection instructions (Nanjing Jiancheng, Nanjing, China). The HTR1A and DRD2 levels in brain tissue were detected by ELISA according to the instructions of ELISA kits. All the ELISA kits were purchased from Elabscience Biotechnology Co., Ltd.

#### 2.4.5. Statistics Analysis

All data were expressed as the mean ± standard deviation (SD). GraphPad Prism 5.01 software was used to ascertain statistically significant differences. The differences among multiple groups were evaluated using the one-way analysis of variance (ANOVA), and the *t*-test was used for pairwise comparison. The difference between the means was considered to be statistically significant at *P* < 0.05 and *P* < 0.01.

## 3. Results and Discussion

### 3.1. UPLC-UESI-Q Exactive Focus Analysis of TTNC Extracts

**Figure 2:**
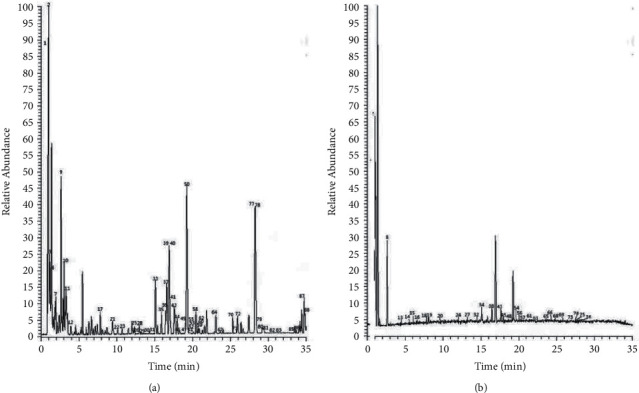
The total ion flow diagram of TTN. (a) The positive ion mode. (b) The negative ion mode.

In terms of mapping the chemical profiles of the extracts of TTNC, data of mass fragmentation coupled with high-resolution spectrometry provided sufficient information. In addition, UPLC-UESI-Q Exactive Focus was used in this present study. The base peak chromatogram (BPC) of TTNC extracts in the mode of positive and negative ions is shown in Figure 2. Compounds were identified by determining the elemental compositions of the precursor and product ions. The molecular formula and rational fragmentation patterns and pathways of these compounds were then identified based on a comparison of these data with chemical databases. According to this situation, the UPLC-UESI-Q Exactive Focus method was used in combination with available databases and literature data. According to ChemSpider (https://www.chemspider.com), the mass spectrometry database (https://www.massbank.jp), and many related Chinese herbal medicine research chemical components [26–38], the chemical database was established to identify the components of TTNC. The source of chemical components in TTNC was determined by the sample injection analysis of 6 botanical drugs and the ion flow extraction from the first-level high-resolution data. Finally, a total of 88 compounds were obtained, including 29 ingredients in Angelicae Sinensis Radix, 25 ingredients in Polygoni Multiflori Radix, 13 ingredients in Gastrodiae Rhizoma, 12 ingredients in Saposhnikoviae Radix, 8 ingredients in Smilacis Glabrae Rhizoma, and 1 ingredient in Scorpio. The results are given in Table S1 in Supplemental Materials.

### 3.2. The Targets of the Chemical Composition for TTNC in the Treatment of Migraine

**Figure 3:**
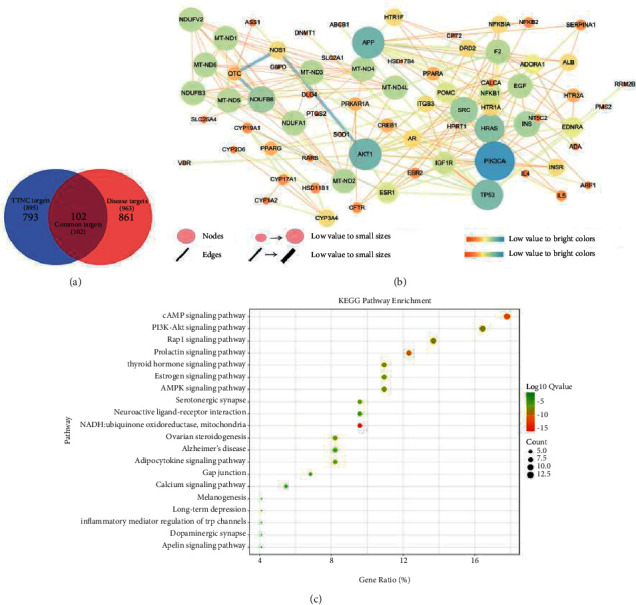
The results of Venn analysis, protein-protein interaction (PPI), and the KEGG pathway enrichment analysis. (a) The Venn's diagrams between the component targets for TTNC and disease targets for migraine. (b) The PPI network for TTNC in treatment with migraine. (c) The results of KEGG pathway enrichment analysis for TTNC in treatment with migraine.

To obtain the targets of the 88 components for TTNC, target fishing was performed in accordance with the method described in Section 2.2.1. As a result, 895 targets were obtained for 50 compounds. Then, “migraine” was entered as keywords, and there were 2759 targets obtained from the GeneCards, and 963 targets were considered as the migraine targets according to relevance score. An analysis was performed using Venn online software to identify common targets between component targets and disease targets with Venn's diagrams (Figure 3(a)). Finally, 102 targets were determined as the potential targets of TTNC in treating migraine.

### 3.3. The PPI Network Construction and Core Targets Determination

The 102 targets of TTNC were introduced into the STRING 11.0 platform for searching the interaction between potential targets (i.e., targets of TTNC). The interaction results were introduced into the Cytoscape 3.7.2 software to draw the PPI network. Also, the nodes represented targets and the edges represented the interaction between different nodes. After removing the targets far away from the main network, there were 73 nodes and 206 edges (Figure 3(b)). The larger the circle, the brighter the color. This indicates that it plays a more important role in the network, and it will be more crucial for the occurrence of migraine and the treatment of TTNC.

### 3.4. The Results of KEGG Pathway Analysis

The 73 targets were mapped to a total of 124 KEGG pathways. The obviously unrelated KEGG pathways were removed, such as “pathways in cancer,” “prostate cancer,” “hepatitis C,” and so on. Finally, the pathways with the top 20 KEGG pathways of the log *q*-value value are shown in Figure 3(c). According to the results, the cAMP signaling pathway, PI3K-Akt signaling pathway, rap1 signaling pathway, and NADH: ubiquinone oxidoreductase, mitochondria may be the important pathways for TTNC. In these pathways, the cAMP signaling pathway was one of the most important pathways with the most enriched targets (12 targets, including ADORA1, AKT1, CFTR, CREB1, DRD2, EDNRA, HTR1A, HTR1F, NFKB1, NFKBIA, PIK3CA, and PPARA) and smaller log *q*-value (log *q*-value = −1.04*E* + 01). The number of enriched targets in the PI3K-Akt signaling pathway was 12. The log *q*-value is −8.52*E* + 00. In addition, NADH: ubiquinone oxidoreductase, mitochondria have the smallest log *q*-value (log *q*-value = −1.55*E* + 01), and may be one of the other most important pathways for TTNC.

### 3.5. The Herbs-Compounds-Targets-Pathways (H-C-T-P) Network Construction and Analysis

**Figure 4:**
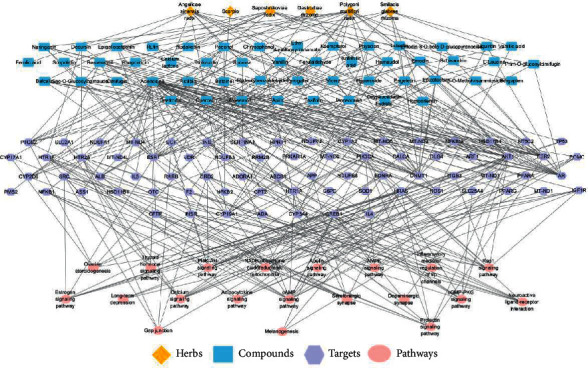
The herbs-compounds-targets-pathways (H-C-T-P) network for TTNC in treatment with migraine.

The H-C-T-P network was constructed, and analysis for TTNC in treatment with migraine was done by using Cytoscape 3.7.2 (Figure 4). The network contained 150 nodes (6 herbs, 51 compounds, 73 targets, and 20 pathways) and 381 edges. In Figure 4, the herbs nodes are shown in yellow diamond, the compounds nodes are shown in blue hexagon, the targets nodes are shown in purple rectangle, and the pathways nodes are shown in pink ellipse. The gray edges represented the relationships among the herb, compounds, targets, and pathways. The topological properties of the network were analyzed by the network analyzer plugin in Cytoscape 3.7.2, including degree centrality (DC), betweenness centrality (BC), and closeness centrality (CC) (Tables 1–3). These topological properties are important parameters of network analysis, which can reflect the importance of each node in the network. The higher the topological properties, the more important the compound or target is in the network.

According to the results, we obtained 18 potential key compounds, 20 potential key targets, and 6 potential key pathways with higher than the average of DC. In the key compounds, gastrodin, the main effective constitute from Gastrodiae Rhizoma, has been indicated for migraine treatment and prophylaxis more than 30 years, with demonstrated safety [39]. The research of Wang et al. showed that the antimigraine effect of gastrodin is associated with inhibition of the trigeminal pain pathway at peripheral and central sites in the model of nitroglycerin (NTG)-induced migraine in rats. The underlying mechanism of gastrodin reflected by a decrease in c-Fos expression in TNC, direct scavenging of NO, and decrease in the subsequent peripheral release of CGRP [40]. In addition, adenosine is a purine nucleoside that plays a critical role in numerous cellular and molecular functions, such as the metabolism, cell signaling, purinergic neuronal signaling, and inflammation, throughout the brain. Recent studies have proved that the role of adenosine in different forms of headache, headache triggers, and basic headache physiology and suggested it was a core component to headache pain. In addition, adenosine signaling may initiate headache pain by modulation of intracellular cAMP production or AMPK activity that can change neuronal conductance within critical trigeminal pain processing brain regions [41]. Besides, some components of Saposhnikoviae Radix have also showed higher DC, BC, and CC, such as nodakenin, sec-O-glucosylhamaudol, 5-O-methylvisammioside, and prim-O-glucosylcimifugin. Saposhnikoviae Radix mainly has antipyretic, analgesic, sedative, anti-inflammatory, antibacterial, antitumor, improving immune function, antiallergy, anticoagulant, and other pharmacological effects. It is one of the commonly used medicines for headache.

In terms of the key targets, AKT1 is one of 3 closely related serine/threonine-protein kinases (AKT1, AKT2, and AKT3) called the AKT kinase, which regulates many processes including the metabolism, proliferation, cell survival, growth, and angiogenesis through serine and/or threonine phosphorylation of a range of downstream substrates [42–44]. Previous studies have revealed that the expression level of phosphorylated AKT (P-AKT) was significantly increased in a rat model group of migraine compared with the control group via measuring the p-AKT level [45]. Moreover, AKT controlled the tempo of the process of newborn neurons integration during adult neurogenesis, including correct neuron positioning, dendritic development, and synapse formation, and plays a role of key modulator in the AKT-mTOR signaling pathway [46]. MAPK8 and MAPK1 were two of member of MAPK family, and activation of MAPK is suggested to mediate the synthesis and the release of calcitonin gene-related peptide (CGRP) which has long been implicated in the pathophysiology of migraine [47, 48]. Also, they are monoclonal antibodies (mAbs) targeting CGRP or its receptor was approved to be migraine-specific treatment in late decades [49–51]. Thus, the result in this study proved that key targets MAPK1 and MAPK8 regulating migraine through MAPK signaling are consistent with previous research. It was well known that migraine is a nervous system disease with a significant genetic predisposition [52–54]. Furthermore, sufficient studies have proved the significant role of sex hormones in migraine, and variants in the ESR1 gene were linked to migraine. It showed the association between rs2234693 in ESR1 gene and menstrually related migraine in research which contains a cohort of 533 controls and 494 migraine patients [55].

Amounts of evidences supported the significance role of the PI3K-Akt signaling pathway in migraine [45, 56]. A study has confirmed that glucagon-like peptide-1 receptor (GLP-1R) agonist inhibited the upregulation of PI3K/p-Akt in the trigeminal nucleus caudalis (TNC) via Western blotting, which indicated that microglial GLP-1R activation in TNC may suppress the central sensitization of chronic migraine by regulating TNC microglial activation via the PI3K/Akt pathway [57]. Furthermore, PRKCA, as a core target of our research in both the P13K-AKT pathway and MAPK signaling pathway, is calcium-activated and phospholipid and diacylglycerol (DAG)-dependent serine/threonine-protein kinase activating signaling cascade involving MAPK1/3 (ERK1/2) and RAP1GAP, thus promoting cell growth by phosphorylating and activating RAF1. It mediates the activation of the MAPK/ERK signaling cascade and/or upregulates CDKN1A, which facilitates active cyclin-dependent kinase (CDK) complex formation in glioma cells.

### 3.6. Molecular Docking Results

**Figure 5:**
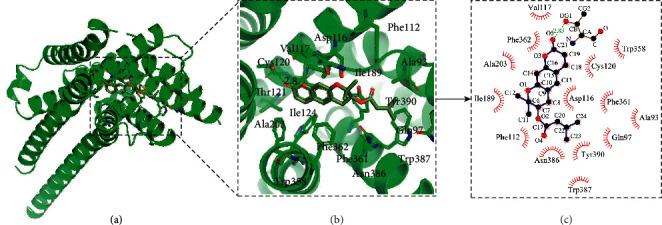
The molecular docking results of TTNC in treatment with migraine.

The molecular docking was carried out to further explore the interaction between some compounds and targets. We selected some key targets that have been reported to be closely related to migraine (DRD2, HTR1A, HTR2A, ESR1, ESR2, CYP19A1, and AR) and the corresponding compounds for molecular docking. The binding energy is given in Table 4. The results of binding energy showed that the interaction between these targets and related compounds was strong. Take the binding between HTR1A and decursin as an example (Figure 5); decursin can bind in the active pocket composed of residues Val117, Phe362, Ala203, Ile189, Phe112, Asn386, Tyr390, Asp116, Cys120, and Trp358 for HTR1. There is a hydrogen bond between Thr121 and decursin, and the binding energy is −8.65 kcal/mol.

### 3.7. Experimental Verification Results

#### 3.7.1. Behavioral Changes

**Figure 6:**
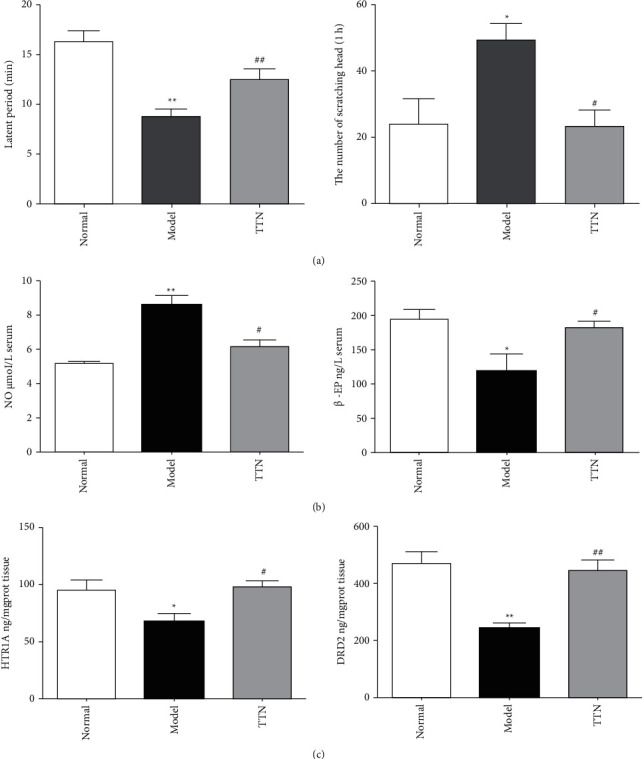
The results of experimental verification. (a) Behavioral changes of the control, model, and TTN group. (b) The contents of NO and *β*-EP in blood serum of the control, model, and TTN group. (c) The HTR1A and DRD2 levels in brain tissue detected by ELISA of the control, model, and TTN group.

As shown in Figure 6(a), compared with the control group, the incubation period of red ears and frequent head scratching in the model group was significantly shortened. In contrast, the number of head scratching was significantly increased within 1 h after nitroglycerin injection (*P* < 0.05). Compared with the model group, the incubation period was significantly prolonged, and the number of head scratching was significantly decreased in the TTNC group (*P* < 0.05). The results showed that TTNC can improve the behavioral changes of migraine rats.

#### 3.7.2. The Content Detection of NO and *β*-EP in Blood Serum

Figure 6(b) shows the content detection results of NO and *β*-EP in blood serum. The content of NO in the animal serum model group increased significantly compared with the control group (*P* < 0.05), indicating that the model was successful. Compared with the model group, the content of NO in the TTNC group was significantly decreased (*P* < 0.05). It is well known that NO plays a key role in the mechanism of migraine and other vascular headache. NO can expand blood vessels, result in neurogenic inflammation, activate the sensitivity of injured sensory neurons, and mediate pain signal transmission of the body and lead to migraine. The experiment in the present study is a classic migraine model. After injection of nitroglycerin, the content of NO in animal blood increase rapidly, resulting in migraine. In the TTNC group, the content of NO decreased significantly, indicating that TTNC can downregulate the production of NO induced by nitroglycerin, so as to alleviate the attack of migraine. In addition, the content of *β*-EP in the model group decreased significantly compared with the control group (*P* < 0.05), while the content of *β*-EP in the TTNC group increased significantly compared with the model group (*P* < 0.05). *β*-EP is *α*-endogenous opioid peptide with a strong analgesic effect. In the case of disorders of the endorphin system in the body, *β*-EP release decreased immediately, the pain threshold of body also decreased, and migraine are more likely to occur. In this experiment, NO induced endogenous opioid peptide system disorder, and the content of *β*-EP decreased. After the intervention of TTNC, the content of *β*-EP increased, indicating that TTNC could upregulate the level of *β*-EP induced by nitroglycerin, so as to relieve migraine. These results showed that TTNC has a good effect on migraine induced by nitroglycerin.

#### 3.7.3. The HTR1A and DRD2 Levels in Brain Tissue Detected by ELISA

The two key targets of HTR1A and DRD2 in brain tissue were verified in vivo by ELISA detection (Figure 6(c)) for the sake of verifying the results of previous prediction after comprehensively considering the results of molecular docking. The results showed that the level of HTR1A of the model group was significantly lower than that of the control group (*P* < 0.05), and the level was significantly higher in the TTNC group compared with the model group (*P* < 0.05). Besides, the level of DRD2 in brain tissue of the model group was significantly lower than that of the control group (*P* < 0.01), and compared with the model group, the level of DRD2 treated with TTNC improved significantly (*P* < 0.01).

## 4. Conclusion

In the present study, a systematical research strategy was adopted to investigate the mechanism of TTNC in treatment with migraine. It contained UPLC-UESI-Q Exactive Focus, network pharmacology, and in vivo experiment. Also, the target fishing, protein-protein interaction (PPI), KEGG pathway enrichment analysis, and herbs-compounds-targets-pathways (H-C-T-P) network construction were performed according to the network pharmacology to predict the potential pharmacodynamic components, targets, and pathways. After 18 potential key compounds, 20 potential key targets and 6 potential signaling pathways were obtained for TTNC in treatment with migraine. Finally, molecular docking and experimental were carried out to verify some key targets. More experimental verification and research still need to be carried out for TTNC in treatment with migraine. The results of this study may provide a theoretical basis for further research on the molecular mechanism of TTNC in the treatment of migraine.

## Figures and Tables

**Table 1:** The degree centrality (DC), betweenness centrality (BC), and closeness centrality (CC) values of the key compounds for TTNC in treating migraine.

No.	Compound name	Source	CC	DC	BC
1	Adenosine	Gastrodiae Rhizoma	0.4257	43	0.4103
2	Paeonol	Angelicae Sinensis Radix	0.3326	12	0.0750
3	Resveratrol	Smilacis Glabrae Rhizoma	0.3435	9	0.0334
4	Emodin	Polygoni Multiflori Radix	0.3501	7	0.0374
5	Physcion	Polygoni Multiflori Radix	0.2792	7	0.0231
6	Astilbin	Smilacis Glabrae Rhizoma	0.3468	6	0.0111
7	Baicalin	Angelicae Sinensis Radix	0.3623	6	0.0333
8	Engeletin	Smilacis Glabrae Rhizoma	0.3468	6	0.0111
9	Epicatechin	Polygoni Multiflori Radix	0.3468	6	0.0101
10	Epigallocatechin	Polygoni Multiflori Radix	0.3468	6	0.0101
11	Ferulaldehyde	Angelicae Sinensis Radix	0.2961	6	0.0161
12	Homoorientin	Smilacis Glabrae Rhizoma	0.3341	6	0.0078
13	Kaempferol	Polygoni Multiflori Radix	0.3468	6	0.0101
14	Luteolin	Polygoni Multiflori Radix	0.3468	6	0.0101
15	Naringenin	Polygoni Multiflori Radix	0.3468	6	0.0101
16	Quercetin	Angelicae Sinensis Radix	0.3518	6	0.0282
17	Taxifolin	Smilacis Glabrae Rhizoma	0.3372	6	0.0061
18	Vanillin	Angelicae Sinensis Radix	0.3587	6	0.0290

**Table 2:** The degree centrality (DC), betweenness centrality (BC), and closeness centrality (CC) values of the key targets for TTNC in treating migraine.

No.	Gene name	Target name	CC	DC	BC
1	AKT1	AKT serine/threonine kinase 1	0.4345	24	0.1875
2	ESR1	Estrogen receptor 1	0.3544	20	0.0592
3	ESR2	Estrogen receptor 2	0.3427	18	0.0394
4	AR	Androgen receptor	0.3318	18	0.0887
5	DNMT1	DNA methyltransferase 1	0.3862	18	0.1141
6	HRAS	HRas proto-oncogene, GTPase	0.3596	11	0.0450
7	CYP19A1	Cytochrome P450 family 19 subfamily a member 1	0.2967	10	0.0123
8	NOS1	Nitric oxide synthase, brain	0.3782	10	0.0583
9	HTR1F	5-Hydroxytryptamine receptor 1F	0.2908	9	0.0112
10	HTR2A	5-Hydroxytryptamine receptor 2A	0.2920	9	0.0192
11	PIK3CA	Phosphatidylinositol 4,5-bisphosphate 3-kinase catalytic subunit alpha isoform	0.3510	9	0.0241
12	PTGS2	Prostaglandin G/H synthase 2	0.3349	9	0.0471
13	CREB1	Cyclic AMP-responsive element-binding protein 1	0.3460	8	0.0187
14	HTR1A	5-Hydroxytryptamine receptor 1A	0.3042	8	0.0220
15	INS	Insulin	0.2920	7	0.0089
16	SRC	Proto-oncogene tyrosine-protein kinase Src	0.3333	7	0.0160
17	DRD2	D(2) dopamine receptor	0.3364	6	0.0205
18	EDNRA	Endothelin-1 receptor	0.3160	6	0.0127
19	IGF1R	Insulin-like growth factor 1 receptor	0.3318	6	0.0117
20	INSR	Insulin receptor	0.3333	6	0.0105

**Table 3:** The degree centrality (DC), betweenness centrality (BC), and closeness centrality (CC) values of the key pathways for TTNC in treating migraine.

No.	Pathway	Targets	CC	DC	BC
1	cAMP signaling pathway	ADORA1, AKT1, CFTR, CREB1, DRD2, EDNRA, HTR1A, HTR1F, NFKB1, NFKBIA, PIK3CA, PPARA	0.3436	12	0.0524
2	Retrograde endocannabinoid signaling	ND1, ND2, ND3, ND4, ND4L, ND5, ND6, NDUFA1, NDUFB3, NDUFB8, NDUFV2, PTGS2	0.3085	12	0.0321
3	PI3K-Akt signaling pathway	AKT1, CREB1, EGF, HRAS, IGF1R, IL4, INS, INSR, ITGB3, NFKB1, PIK3CA, TP53	0.3356	12	0.0394
4	Rap1 signaling pathway	AKT1, DRD2, EGF, HRAS, IGF1R, INS, INSR, ITGB3, PIK3CA, SRC	0.3281	10	0.0246
5	Estrogen signaling pathway	AKT1, CREB1, ESR1, ESR2, HRAS, PIK3CA, SRC	0.3311	8	0.0263
6	cGMP-PKG signaling pathway	ADORA1, AKT1, SLC25A4, CREB1, EDNRA, INS, INSR	0.3208	7	0.0125

**Table 4:** The docking energy results of the complex between key targets and the key compounds for TTNC in treatment with migraine.

Protein name	Gene name	PDB ID	Ligand name	PubChem ID	Binding energy (kcal/mol)
5-Hydroxytryptamine receptor 1A	HTR1A	7E2Z	Decursin	442126	−8.65
5-Hydroxytryptamine receptor 2A	HTR2A	6WGT	Hamaudol	164722	−7.94
D(2) dopamine receptor	DRD2	6CM4	Adenosine	60961	−6.32
Nitric oxide synthase, brain	NOS1	3HSN	Adenosine	60961	−5.06
Estrogen receptor	ESR1	5FQP	Epicatechin	72276	−7.70
Estrogen receptor beta	ESR2	2YLY	Epigallocatechin	72277	−6.74
Aromatase	CYP19A1	3S79	Kaempferol	5280863	−6.88
Androgen receptor	AR	2PIW	Paeonol	11092	−4.51

## Data Availability

The data used to support the findings of this study are included within the article and Supplementary Materials.

## Supplementary Materials

Supplementary Materials Table S1: the information of 88 ingredients of TTNC by UPLC-UESI-Q Exactive Focus analysis.
