# Supplementary material for: Identification of Constituents and Exploring the Mechanism for Toutongning Capsule in the Treatment of Migraine
Source: Evid Based Complement Alternat Med. 2022 Jan 15;2022:5528845. doi: 10.1155/2022/5528845 (PMC8783712; doi:10.1155/2022/5528845)
Supplement: Supplementary Materials — Table S1: the information of 88 ingredients of TTNC by UPLC-UESI-Q Exactive Focus analysis. [file 5528845.f1.docx]

**Identification of Constituents and Exploring the Mechanism for Toutongning Capsule in the Treatment of Migraine**

Xia Du^1, 2#^, Zhibiao Di^1#^, Yang Liu^1^, Wenbing Zhi^1^, Yuan Liu^1^, Feng Liu^3,4*^, Hong Zhang^1*^

^1^Institute of Traditional Chinese Medicine, Shaanxi Academy of Traditional Chinese Medicine, Xi'an, Shaanxi, 710003, China

^2^Center for Post-doctoral Studies, China Academy of Chinese Medical Sciences, Beijing, 100700, China

^3^Shaanxi Institute of International Trade &Commence, Xi’an 712046, China

^4^Shaanxi Buchang Pharmaceutical Co. Ltd., Xi’an 710075, China

Correspondence should be addressed to Hong Zhang; [zhanghong919919@163.com](mailto:zhanghong919919@163.com) and Feng Liu; [liufeng1720@163.com](mailto:liufeng1720@163.com)

^#^ Contribute equally.

Table S1. Identification of chemical constituents of TTN by UPLC-UESI-Q Exactive Focus

| No | Target Name | +/- | Area | RT (Meas) | Formula | Expected m/z | Measured m/z | Error（ppm） | Source |
| --- | --- | --- | --- | --- | --- | --- | --- | --- | --- |
| 1 | Betaine | + | 1.050E+09 | 0.98 | C5H11NO2 | 118.0863 | 118.0861 | -1.37 | Scorpio |
| 2 | Sucrose | + | 3.330E+09 | 1 | C12H22O11 | 365.1054 | 365.1050 | -1.23 | Angelicae sinensis radix |
| 3 | L-Leucine | + | 1.980E+08 | 1.02 | C6H13NO2 | 132.1019 | 132.1018 | -0.67 | Angelicae sinensis radix |
| 4 | Epigallocatechin | - | 1.740E+06 | 1.08 | C15H14O7 | 351.0722 | 351.0735 | 3.95 | Polygoni multiflori radix |
| 5 | Shikimic acid | - | 6.690E+07 | 1.11 | C7H10O5 | 173.0456 | 173.0452 | -2.22 | Smilacis glabrae rhizoma |
| 6 | Nicotinic acid | + | 1.480E+08 | 1.34 | C6H5NO2 | 124.0393 | 124.0393 | -0.38 | Gastrodiae rhizoma |
| 7 | Adenosine | + | 1.520E+08 | 2.23 | C10H13N5O4 | 268.1040 | 268.1039 | -0.44 | Gastrodiae rhizoma |
| 8 | Gallic acid | - | 7.790E+07 | 2.49 | C7H6O5 | 169.0142 | 169.014 | -1.71 | Polygoni multiflori radix |
| 9 | Gastrodin | + | 2.170E+09 | 2.65 | C13H18O7 | 309.0945 | 309.0941 | -1.24 | Gastrodiae rhizoma |
| 10 | Schisandrin | + | 1.350E+06 | 2.69 | C24H32O7 | 455.2040 | 455.2052 | 2.66 | Polygoni multiflori radix |
| 11 | 5-Hydroxymethylfurfural | + | 7.890E+08 | 3.3 | C6H6O3 | 127.0390 | 127.0389 | -0.77 | Angelicae sinensis radix |
| 12 | Pyrogallol | + | 7.890E+08 | 3.3 | C6H6O3 | 127.0390 | 127.0389 | -0.77 | Polygoni multiflori radix |
| 13 | Protocatechuic acid | - | 4.030E+07 | 4.45 | C7H6O4 | 153.0193 | 153.0191 | -1.48 | Gastrodiae rhizoma |
| 14 | Vanillin | - | 1.390E+07 | 5.34 | C8H8O3 | 153.0546 | 153.0546 | -0.06 | Angelicae sinensis radix |
| 15 | Syringic acid | - | 1.780E+06 | 5.46 | C9H10O5 | 197.0456 | 197.0455 | -0.32 | Gastrodiae rhizoma |
| 16 | 4-Hydroxybenzoic acid | - | 1.360E+08 | 5.87 | C7H6O3 | 137.0244 | 137.0242 | -1.94 | Gastrodiae rhizoma |
| 17 | Parishin E | + | 4.290E+08 | 7.82 | C19H24O13 | 483.1109 | 483.1107 | -0.4 | Gastrodiae rhizoma |
| 18 | Vanillic acid | - | 3.500E+07 | 7.88 | C8H8O4 | 167.0350 | 167.0347 | -1.87 | Smilacis glabrae rhizoma |
| 19 | p-Hydroxybenzaldehyde | - | 2.880E+08 | 8.1 | C7H6O2 | 121.0295 | 121.0292 | -2.12 | Gastrodiae rhizoma |

Continued Table S1. Identification of chemical constituents of TTN by UPLC-UESI-Q Exactive Focus

| 20 | Isoferulic acid | - | 4.160E+07 | 9.45 | C10H10O4 | 239.0561 | 239.0558 | -1.48 | Gastrodiae rhizoma |
| --- | --- | --- | --- | --- | --- | --- | --- | --- | --- |
| 21 | Chlorogenic acid | + | 6.310E+07 | 9.54 | C16H18O9 | 377.0843 | 377.0841 | -0.59 | Angelicae sinensis radix |
| 22 | Liquiritin | + | 2.500E+06 | 10.05 | C21H22O9 | 441.1156 | 441.1157 | 0.14 | Smilacis glabrae rhizoma |
| 23 | Vanillin | + | 3.790E+07 | 10.46 | C8H8O3 | 153.0546 | 153.0546 | -0.06 | Angelicae sinensis radix |
| 24 | Chrysophanol 8-O-β-D-glucoside | - | 1.810E+07 | 11.92 | C21H20O9 | 439.1000 | 439.0997 | -0.64 | Gastrodiae rhizoma |
| 25 | Parishin C | + | 1.350E+08 | 12.29 | C32H40O19 | 751.2056 | 751.2051 | -0.61 | Gastrodiae rhizoma |
| 26 | Paeonol | + | 2.580E+06 | 12.54 | C9H10O3 | 167.0703 | 167.0703 | 0.07 | Angelicae sinensis radix |
| 27 | Epicatechin | - | 2.010E+08 | 12.96 | C15H14O6 | 335.0772 | 335.0766 | -1.82 | Polygoni multiflori radix |
| 28 | Scopoletin | + | 2.960E+07 | 13.31 | C10H8O4 | 215.0315 | 215.0314 | -0.57 | Saposhnikoviae radix |
| 29 | Isofraxidin | + | 2.340E+07 | 13.87 | C11H10O5 | 245.0420 | 245.0419 | -0.69 | Saposhnikoviae radix |
| 30 | Ligusticolide G | + | 1.920E+06 | 14.04 | C12H16O3 | 207.1027 | 207.1023 | -1.59 | Angelicae sinensis radix |
| 31 | Ferulic acid | + | 2.540E+07 | 14.16 | C10H10O4 | 195.0652 | 195.0652 | -0.02 | Angelicae sinensis radix |
| 32 | Vicine | - | 1.910E+08 | 14.29 | C10H16N4O7 | 339.0713 | 339.0714 | 0.42 | Polygoni multiflori radix |
| 33 | Prim-O-glucosylcimifugin | + | 8.940E+08 | 15.08 | C22H28O11 | 491.1524 | 491.1519 | -1.07 | Saposhnikoviae radix |
| 34 | Parishin A | - | 8.210E+07 | 15.33 | C45H56O25 | 995.3038 | 995.3023 | -1.49 | Gastrodiae rhizoma |
| 35 | (E)-2,3,5,4 '-tetrahydroxy styrene-2-O - β-D-glucoside | + | 3.840E+08 | 15.85 | C20H22O9 | 405.1191 | 405.1183 | -1.96 | Polygoni multiflori radix |
| 36 | Ethyl 4-methoxycinnamate | + | 2.100E+08 | 16.58 | C12H14O3 | 207.1016 | 207.1014 | -0.77 | Angelicae sinensis radix |
| 37 | Ligusticolide I | + | 7.510E+06 | 16.6 | C12H16O4 | 225.1121 | 225.1121 | -0.08 | Angelicae sinensis radix |
| 38 | Cinnamic acid | - | 2.560E+07 | 16.69 | C9H8O2 | 193.0506 | 193.0502 | -1.98 | Angelicae sinensis radix |
| 39 | Nodakenin | + | 1.020E+07 | 16.82 | C20H24O9 | 431.1312 | 431.1308 | -1.12 | Saposhnikoviae radix |

Continued Table S1. Identification of chemical constituents of TTN by UPLC-UESI-Q Exactive Focus

| 40 | Taxifolin | + | 3.870E+08 | 16.92 | C15H12O7 | 305.0656 | 305.0652 | -1.19 | Smilacis glabrae rhizoma |
| --- | --- | --- | --- | --- | --- | --- | --- | --- | --- |
| 41 | Astilbin | + | 1.900E+09 | 16.92 | C21H22O11 | 473.1054 | 473.1050 | -0.82 | Smilacis glabrae rhizoma |
| 42 | Cimifugin | + | 2.950E+08 | 17.06 | C16H18O6 | 329.0996 | 329.0991 | -1.33 | Saposhnikoviae radix |
| 43 | Rhaponticin | - | 5.600E+06 | 17.9 | C21H24O9 | 419.1348 | 419.1341 | -1.59 | Polygoni multiflori radix |
| 44 | Hyperoside | + | 5.610E+05 | 18.22 | C21H20O12 | 487.0847 | 487.0836 | -2.16 | Polygoni multiflori radix |
| 45 | Apiin | - | 1.010E+06 | 18.31 | C26H28O14 | 609.1461 | 609.1450 | -1.79 | Polygoni multiflori radix |
| 46 | Rutin | + | 1.270E+06 | 18.32 | C27H30O16 | 633.1426 | 633.1432 | 1.01 | Polygoni multiflori radix |
| 47 | Polygoni multiflori radix b | + | 1.300E+06 | 18.32 | C21H22O9 | 419.1337 | 419.1337 | 0.09 | Polygoni multiflori radix |
| 48 | Azelaic acid | - | 4.350E+07 | 18.33 | C9H16O4 | 187.0976 | 187.0972 | -1.79 | Angelicae sinensis radix |
| 49 | Engeletin | + | 3.270E+08 | 19.07 | C21H22O10 | 457.1105 | 457.1099 | -1.37 | Smilacis glabrae rhizoma |
| 50 | 5-O-Methylvisammioside | + | 3.210E+09 | 19.22 | C22H28O10 | 475.1575 | 475.1568 | -1.48 | Saposhnikoviae radix |
| 51 | Oxypeucedanin hydrate | + | 2.950E+06 | 19.36 | C16H16O6 | 327.0839 | 327.0834 | -1.55 | Angelicae sinensis radix |
| 52 | 2,5-dimethyl-7-hydroxychromonone | + | 1.030E+07 | 19.36 | C11H10O3 | 191.0703 | 191.0702 | -0.5 | Polygoni multiflori radix |
| 53 | Trans ferulic tyramine | + | 2.570E+06 | 19.46 | C18H19NO4 | 314.1387 | 314.1383 | -1.29 | Polygoni multiflori radix |
| 54 | (-)-Gallocatechin | - | 1.590E+06 | 19.79 | C15H14O7 | 305.0667 | 305.0664 | -0.9 | Polygoni multiflori radix |
| 55 | Quercetin | + | 5.910E+06 | 19.8 | C15H10O7 | 303.0499 | 303.0497 | -0.92 | Angelicae sinensis radix |
| 56 | Homoorientin | - | 4.280E+07 | 19.81 | C21H20O11 | 447.0933 | 447.0925 | -1.75 | Smilacis glabrae rhizoma |
| 57 | Resveratrol | - | 5.840E+06 | 19.86 | C14H12O3 | 227.0714 | 227.0710 | -1.43 | Smilacis glabrae rhizoma |
| 58 | Ferulaldehyde | + | 3.770E+06 | 20.38 | C10H10O3 | 179.0703 | 179.0702 | -0.53 | Angelicae sinensis radix |
| 59 | Psoralen | + | 1.090E+08 | 20.55 | C11H6O3 | 187.0390 | 187.0389 | -0.16 | Angelicae sinensis radix |
| 60 | Isopsoralen | + | 1.090E+08 | 20.55 | C11H6O3 | 187.039 | 187.0389 | -0.16 | Angelicae sinensis radix |

Continued Table S1. Identification of chemical constituents of TTN by UPLC-UESI-Q Exactive Focus

| 61 | Chrysophanol | - | 6.710E+07 | 20.73 | C15H10O4 | 253.0506 | 253.0503 | -1.15 | Polygoni multiflori radix |
| --- | --- | --- | --- | --- | --- | --- | --- | --- | --- |
| 62 | Bergapten | + | 7.850E+07 | 20.74 | C12H8O4 | 217.0495 | 217.0495 | -0.33 | Saposhnikoviae radix |
| 63 | Naringenin | - | 2.890E+07 | 21.89 | C15H12O5 | 271.0612 | 271.0607 | -1.85 | Polygoni multiflori radix |
| 64 | Sec-O-Glucosylhamaudol | + | 1.470E+08 | 23.02 | C21H26O10 | 461.1418 | 461.1415 | -0.74 | Saposhnikoviae radix |
| 65 | Aloeemodin | - | 3.830E+06 | 23.2 | C15H10O5 | 271.0601 | 271.0597 | -1.5 | Polygoni multiflori radix |
| 66 | Emodin | - | 3.830E+06 | 23.2 | C15H10O5 | 271.0601 | 271.0597 | -1.5 | Polygoni multiflori radix |
| 67 | Angelicae sinensis radix Ketone | + | 7.070E+05 | 23.48 | C16H16O5 | 289.1070 | 289.1067 | -1.14 | Angelicae sinensis radix |
| 68 | Emodin-8-O-beta-D-glucopyranoside | - | 8.900E+07 | 24.19 | C21H20O10 | 431.0984 | 431.0975 | -2.01 | Polygoni multiflori radix |
| 69 | 4-(4-hydroxybenzoyl) benzyl alcohol | - | 8.280E+06 | 25.26 | C14H14O3 | 229.0870 | 229.0865 | -2.26 | Gastrodiae rhizoma |
| 70 | Senkyunolide A | + | 1.530E+08 | 25.32 | C12H16O2 | 193.1223 | 193.1222 | -0.53 | Angelicae sinensis radix |
| 71 | Hamaudol | + | 2.280E+07 | 25.58 | C15H16O5 | 439.1599 | 439.1596 | -0.67 | Saposhnikoviae radix |
| 72 | Emodin methyl ether -8-O- mine -D- pyrine glucoside | + | 1.590E+08 | 25.8 | C22H22O10 | 447.1286 | 447.1284 | -0.27 | Polygoni multiflori radix |
| 73 | Physcion | - | 6.180E+06 | 26.39 | C16H12O5 | 283.0612 | 283.0605 | -2.31 | Polygoni multiflori radix |
| 74 | Kaempferol | - | 5.920E+06 | 26.83 | C15H10O6 | 287.055 | 287.0544 | -1.97 | Polygoni multiflori radix |
| 75 | Luteolin | - | 5.920E+06 | 26.83 | C15H10O6 | 287.0550 | 287.0544 | -1.97 | Polygoni multiflori radix |
| 76 | omega-hydroxy-emodin | - | 5.920E+06 | 26.83 | C15H10O6 | 287.0550 | 287.0544 | -1.97 | Polygoni multiflori radix |
| 77 | 3-n-Butylphathlide | + | 2.620E+09 | 28.27 | C12H14O2 | 191.1067 | 191.1064 | -1.52 | Angelicae sinensis radix |
| 78 | Ligustilide | + | 2.620E+09 | 28.27 | C12H14O2 | 191.1067 | 191.1064 | -1.52 | Angelicae sinensis radix |
| 79 | Isoimperatorin | + | 4.990E+07 | 28.36 | C16H14O4 | 293.0784 | 293.0780 | -1.46 | Saposhnikoviae radix |
| 80 | 3-Butylidenephthalide | + | 2.840E+08 | 29.33 | C12H12O2 | 189.0910 | 189.0908 | -1 | Angelicae sinensis radix |

Continued Table S1. Identification of chemical constituents of TTN by UPLC-UESI-Q Exactive Focus

| 81 | Baicalin | + | 6.480E+05 | 29.65 | C21H18O11 | 469.0741 | 469.0727 | -3 | Angelicae sinensis radix |
| --- | --- | --- | --- | --- | --- | --- | --- | --- | --- |
| 82 | Phellopterin | + | 2.510E+06 | 30.01 | C17H16O5 | 301.107 | 301.1069 | -0.39 | Saposhnikoviae radix |
| 83 | Decursin | + | 1.010E+08 | 31.37 | C19H20O5 | 351.1203 | 351.1200 | -0.84 | Saposhnikoviae radix |
| 84 | Cnidium lactone | + | 1.040E+06 | 31.51 | C15H16O3 | 245.1172 | 245.1168 | -1.69 | Angelicae sinensis radix |
| 85 | Guaiacylglycerin | + | 1.370E+06 | 33.55 | C20H24O7 | 375.1449 | 375.1442 | -1.96 | Angelicae sinensis radix |
| 86 | 11S, 16R-dihydroxyoctadeca-9Z, 17-dien-12, 14-diynoic-1-yl acetate | + | 5.600E+07 | 33.96 | C20H28O4 | 331.1915 | 331.1909 | -1.7 | Angelicae sinensis radix |
| 87 | Levistilide A | + | 8.050E+08 | 34.74 | C24H28O4 | 403.1880 | 403.1875 | -1.27 | Angelicae sinensis radix |
| 88 | Caffeic acid | + | 7.590E+06 | 34.84 | C9H8O4 | 179.0350 | 179.0347 | -1.4 | Angelicae sinensis radix |
